# The relationship between physical activity and self-control in college students: the mediating role of trait anxiety

**DOI:** 10.3389/fpubh.2026.1819675

**Published:** 2026-06-03

**Authors:** Xianda Zhang, Fapeng Wang

**Affiliations:** China Football College, Beijing Sport University, Beijing, China

**Keywords:** college students, mediation, physical activity, self-control, trait anxiety

## Abstract

**Purpose:**

To enhance self-control abilities among college students and maintain their mental health, this study investigates the influencing factors and mechanisms of self-control among college students.

**Methods:**

Using convenience sampling, 452 college students were surveyed using the International Physical Activity Questionnaire (short form), the Self-Control Scale, and the Trait Anxiety Scale. Demographic data were collected to explore the factors influencing self-control.

**Results:**

1. Physical activity significantly and positively predicted self-control among college students. 2. Trait anxiety partially mediates the relationship between physical activity and self-control among college students. 3. Gender moderates the relationship between physical activity and trait anxiety.

**Conclusion:**

There is a close relationship among physical activity, self-control, and trait anxiety among college students. Physical activity is directly linked to self-control and is also indirectly linked to self-control through trait anxiety. Furthermore, the relationship between physical activity and trait anxiety is moderated by gender.

## 1 Introduction

The ability to follow one’s feeling of autonomy in controlling one’s instincts, refusing to give in to immediate wants and primal urges, and making an effort to engage in one’s own goal-oriented actions in order to reap long-term rewards is known as self-control (1). A sense of meaning in life (2), self-efficacy (3), higher subjective well-being (4), improved self-esteem (5), and fewer substance abuse and addiction behaviors (6, 7) are all linked to effective self-control. On the other hand, a lack of self-control will result in several detrimental effects, such as increased procrastination and impulsive behavior (8, 9), higher loneliness (10), shyness, and social anxiety (11). It is evident that self-control is crucial for people since it can support them in overcoming obstacles, preserving their mental well-being, and guiding their own development. But the psychological issues that college students are facing have become worse, and this phenomena is directly linked to the lack of self-control among these students (12). As a result, it is now vital to figure out how to help college students become more self-controllable and less likely to develop psychiatric issues.

Physical activity can be an effective way to overcome the self-control deficiencies of college students and improve their mental health. With the social and economic development and the improvement of production technology, the proportion of manual laborers in the current society has gradually declined, and mechanical and repetitive physical labor has gradually been replaced by machines, and the time that individuals spend on mental labor is increasing, which also leads to a gradual decline in the amount of physical activity carried out by individuals in their daily working life. According to the World Health Organization’s report on the main risk factors for death, physical inactivity has become the fourth highest risk factor for death (13, 14). Physical inactivity is also widespread in the college student population, and physical inactivity is especially prominent among female college students. According to the China College Student Health Survey Report released in 2020, most college students are confident in their physical health, but one-tenth of them are still not confident in this. Nearly 30% of college students believe that their exercise is not up to standard, and less than half of college students can insist on exercising more than three times a week, of which 60% of male college students say they can exercise more than three times a week, while less than 40% of female college students say they can reach this standard. In addition, male college students’ assessments of their physical health and mental health are significantly higher than those of female college students (15). The Report on the Development of China’s National Mental Health (2021–2022) pointed out that the detection rate of depression among Chinese college students is 21.48%, and the detection rate of anxiety is 43.28%, with male students scoring higher than female students in depression and female students scoring higher than male students in anxiety. Moreover, exercise has a positive significance in maintaining mental health, and the depression detection rate of the group with zero weekly exercise frequency is much higher than that of other groups (16). The above reports show that the physical and mental health of Chinese college students is not optimistic, and that exercise can help individuals reduce psychological problems and maintain mental health, and mental health is closely related to self-control, so physical activity should help individuals overcome self-control deficits.

### 1.1 Relationship between physical activity and self-control

Any movement of the body caused by skeletal muscles that causes the organism to waste more energy than it would at rest (basal) is considered physical activity (17). Sports and physical exercise are the single manifestations of physical activity in the strictest definition. Whereas, physical activity in the broader sense includes physical activities in daily life in addition to sports and exercise (18), such as occupational activities, household chores, leisure activities, and so on. It has been demonstrated that self-control and physical activity are strongly correlated (19). Self-control and physical activity may be positively correlated, according to some research. When Guo et al. (20) looked at the relationship between self-control and physical activity among Chinese college students, they discovered that self-control was significantly and favorably predicted by physical activity. The study by C. Li et al. (21) found the same outcome. The direction of the relationship between physical exercise and self-control, however, continues to divide some studies. Their primary area of investigation is how various levels of exercise difficulty and intensity affect self-control. Exercise intensity and self-control are thought to have a “inverted U-curve,” meaning that moderate exercise greatly increases self-control while high-intensity exercise may cause self-control depletion, which lowers an individual’s capacity for self-control (47). Some researchers argue that complex physical training consumes a significant amount of self-control resources, leading to self-control depletion and ultimately reducing self-control capacity (22).

To explain this phenomenon, the strength model of self-control provides a robust theoretical framework (23). This theory posits that an individual’s capacity for self-control depends on a finite, renewable psychological resource. Any action requiring active control—including physical activity—depletes this resource, leading to temporary depletion, or a state of “self-exhaustion,” which in turn impairs subsequent self-control performance. However, the model also notes that this resource is similar to muscle strength and can be enhanced through regular training. Persistent engagement in physical activity, particularly the constant overcoming of inertia, fatigue, and external distractions during exercise, can be viewed as a process of “training” self-control resources. Such repetitive practice can increase the “reserves” of self-control resources or improve their utilization efficiency, making individuals less prone to depletion and enabling better performance when executing self-control tasks in the future. Therefore, the question of whether increasing physical activity can effectively enhance an individual’s self-control requires further verification. Based on this, Hypothesis 1 is proposed: Physical activity can significantly and positively predict self-control among college students.

### 1.2 The mediating role of trait anxiety

Anxiety is an unpleasant emotion that people experience in reaction to impending or possibly harmful situations. It includes worry, impatience, and other negative emotions (24). Additionally, trait anxiety is a sort of anxiety that is a reasonably consistent personality characteristic and panic inclination (24). Stress buffer theory suggests that physical activity acts as a stress buffer and reduces the individual’s tendency to react to chronic stress with anxiety by enhancing the regulatory capacity of the physiological stress system (50). Physical activity has been shown to assist people deal with their anxiety, and there is a negative correlation between trait anxiety and physical activity (25–27). Researchers discovered that greater home walking was linked to decreased anxiety levels in a study examining pregnant women’s levels of physical activity throughout pregnancy (28). Xi-Bin et al. (29) investigated the relationship between anxiety and physical activity during home study among high school students amid an epidemic and discovered that moderate to high intensity physical activity can help high school students avoid anxiety symptoms. Using a meta-analysis to summarize the results of previous research on whether physical activity can help individuals ward off symptoms of depression and anxiety, one researcher ultimately found that physical activity can improve depression and anxiety conditions, and that the improvement in depression was more pronounced (30). The Multidimensional Anxiety Theory posits that anxiety consists of two components: cognitive worry and physical tension (31). Physical activity not only helps expend excess stress energy and reduce an individual’s physical tension but also helps divert attention and interrupt catastrophic thinking. Therefore, physical activity is believed to systematically reduce an individual’s baseline anxiety levels.

Self-control and trait anxiety have been found to be negatively correlated (32, 33). According to one study, college students’ self-control at enrollment was an accurate predictor of their anxiety levels 3 years later; more specifically, a higher level of self-control at enrollment was associated with lower levels of anxiety later in life (34). When examining the impact of trait anxiety on secondary school students’ academic performance, Yaohui and Chao (35) discovered that self-control mediated the effects of trait anxiety on academic performance and that there was a significant negative correlation between both. The strength model of self-control posits that posits that the total amount of an individual’s attention and cognitive resources is finite. When individuals perform self-control tasks, they consume these limited cognitive resources. When cognitive resources are depleted, task performance is further impaired (23). Individuals with high trait anxiety expend more resources in daily life to manage diffuse anxiety and worry. This leaves them with insufficient resources for active self-control, thereby impairing their daily performance. Based on this, Hypothesis 2 is proposed: trait anxiety mediates the relationship between physical activity and self-control.

### 1.3 The moderating role of gender

When examining the relationship between physical activity and trait anxiety and self-control among college students, the influence of gender cannot be overlooked. Previous research has shown that there are significant differences in physical activity levels between genders, with men typically engaging in higher levels of physical activity than women (36). For example, Sher et al. (37) found that men spent significantly more time exercising than women. Specifically, men reported exercising 3.41 days in the past week, while women reported exercising 3.21 days. Karavirta et al. (38), in their investigation of physical activity energy expenditure (PAEE) among individuals of different genders, found that men’s PAEE was 41.4 kJ/kg/day, significantly higher than women’s 34.8 kJ/kg/day. Furthermore, there are significant differences in anxiety levels between the genders. Graham (39), in a study of the prevalence of anxiety disorders among Australian residents, found that the prevalence among women was significantly higher than among men. This finding was also observed among South Korean residents (40). Based on this, Hypothesis 3 is proposed: gender moderates the relationship between physical activity and trait anxiety.

In summary, although previous studies have examined the relationships between physical activity, trait anxiety, and self-control in pairs, the overall mechanism of action among these three variables—particularly the mediating role of trait anxiety and the moderating role of gender—remains to be further investigated. Therefore, this study aims to construct a moderated mediation model. Through empirical investigation, it seeks to explore in depth “how” (mediated by trait anxiety) and “when” (moderated by gender) physical activity influences college students’levels of self-control, with the goal of providing more targeted theoretical foundations and practical guidance for enhancing college students’self-control abilities and mental health. Based on this, we propose the following hypotheses:

*H1*: Physical activity significantly and positively predicts self-regulation among college students.

*H2*: Trait anxiety mediates the relationship between physical activity and self-control.

*H3*: Gender moderates the relationship between physical activity and trait anxiety.

## 2 Materials and methods

### 2.1 Participants

In terms of sample size, according to the formula used by Hajime Uchida scholars to calculate sample size: $n\geq {\left(\frac{k}{\alpha }\right)}^{2}P(1-P)$ . In this formula, when *p* = 0.5, *α* = 0.05, k = 1.96, at this time n = 385, which means that at least 385 survey subjects are needed. Convenience sampling was employed to distribute electronic questionnaires at a university in China. QR codes for the electronic questionnaires were printed on gifts, and the questionnaires were distributed by giving away these gifts. A total of 500 gifts were distributed; however, since 20 participants received gifts but did not scan the QR code to complete the questionnaire, 480 valid responses were ultimately collected. After excluding 16 questionnaires with missing values and 12 questionnaires with consistent but non-random responses, 452 valid questionnaires were ultimately retained, resulting in a response rate of 90.4%. The questionnaires with missing values referred to those where the physical activity section was incomplete (the physical activity questions were fill-in-the-blank items, and some students entered only zeros for all answers), while those with consistent but non-random responses referred to students who selected the same option for every question throughout the questionnaire. The university selected for this study is a comprehensive university jointly built by a province and a ministry. Its place of origin covers 31 provinces in China, with 13.4% ethnic minority students, which is close to the national average of 10.01, and 46.2% male students, which is close to the national average of 50% (49), which guarantees the representativeness of the sample’s basic characteristics to a certain extent.

Finally, 452 questionnaires were validly recovered, with an effective recovery rate of 90.4%. There were 166 (36.7%) male students and 286 (63.3%) female students. The age was 20.9 ± 2.2 years. In terms of majors, 66 (14.6%) were in science, 100 (22.1%) in engineering, 40 (8.8%) in agriculture, 80 (17.7%) in medicine, and 166 (36.8%) in humanities and social sciences. In terms of grade, there were 130 (28.8%) freshmen, 136 (30.1%) sophomores, 72 (15.9%) juniors, 24 (5.8%) seniors, and 90 (19.5%) post- graduate students.

### 2.2 Materials

#### 2.2.1 Trait anxiety

The State–Trait Anxiety Inventory (STAI) developed by Spielberg et al. was used. The scale is divided into two subscales: state anxiety and trait anxiety. The STAI was subsequently introduced to China and revised for the Chinese college student population. The translation process did not fundamentally change the items of the scale and basically maintained the original meaning. The Cronbach’s alpha coefficient of the revised state anxiety scale was 0.91, and that of the trait anxiety scale was 0.88 (41). In this study, only the Trait Anxiety Inventory was used, which consists of 20 items, including 11 positively scored items and 9 negatively scored items. A four-point scale, with options ranging from 1 to 4, corresponding to “not at all” to “very much,” was used to assess the stable and frequent emotional state of the subjects, with higher total scores indicating higher levels of trait anxiety. The scale used in this study had a Cronbach’s alpha coefficient of 0.81.

#### 2.2.2 Self-control

The Self-Control Questionnaire (SCS) developed by Bandura and revised by Shu-Hua and Yong-yu (42), was used. The SCS was revised by deleting items with low correlation with the total score (r < 0.3), and modifying items with ambiguous expressions, such as replacing the item “I lose control of myself because of my feelings” with the item “I get so worked up that I can’t hold on to myself because of my emotions”. Items that are obviously inconsistent with life in China were modified, such as “I often use drugs excessively” was changed to “I often drink alcohol or surf the Internet excessively”, and so on. The final scale consisted of 19 items, with a Cronbach’s *α* coefficient of 0.86, and the total correlation of all items ranged from 0.33 to 0.56. The 19 items on the scale are divided into 5 dimensions: concentrating on work, impulse control, healthy habits, avoiding temptation, and controlling entertainment. Each question was answered by participants using a five-point Likert scale. Four items received an adverse score, while 15 items received a positive score. Poorer self-control is indicated by higher overall scores. The scale used in this study had a Cronbach’s alpha coefficient of 0.81.

#### 2.2.3 Physical activity

The short forms of the International Physical Activity Questionnaire (IPAQ-SF) (43) were employed. Seven components made up the scale: walking frequency and duration, moderate and vigorous exercise, and sedentary activity time information. Based on the frequency and time information of different intensity exercise, the overall activity level of an individual for a week was weighted and calculated. Physical activity levels for different intensity exercises were calculated as MET × frequency per week (d/w) × activity time per day (min/d). Walking received a MET of 3.3, moderate-intensity exercise a MET of 4.0, and vigorous-intensity exercise a MET of 8.0. The total activity for a week was the sum of the three exercise intensities.

### 2.3 Data processing and analyzing

All data were analyzed using SPSS 26.0. The data were first standardized, and all subsequent analyses were based on the standardized data. Subsequently, a common method bias test was conducted using the unmeasured latent method construct (ULMC) approach. Using AMOS software, a baseline confirmatory factor analysis (CFA) model was constructed comprising three factors: physical activity, trait anxiety, and self-control. Subsequently, a common method factor was added to the baseline model, and the loadings of all measurement items on this factor were allowed to be freely estimated to construct the common method factor model. The impact of common method bias on the results was assessed by comparing the fit indices of the two models. Finally, after controlling for grade level, major, and physical activity intensity, we used self-control scores as the dependent variable, physical activity scores as the independent variable, and trait anxiety as the mediating variable. We employed Model 4 in the PROCESS 4.1 plugin to test the mediating role of trait anxiety between physical activity and self-control. Subsequently, Model 7 was used to test the moderating effect of gender on the relationship between physical activity and trait anxiety, while controlling for grade level and major.

## 3 Results

### 3.1 Common method bias test

Because the study used self-report methods to collect data, common method bias may be present. Therefore, this study employed the ULMC technique to examine common method bias. The results showed that after incorporating the common method factor, the fit indices of the two models did not change significantly (△RMR = 0.01, △RMSEA = 0.01, △CFI = 0.10, △TLI = 0.09). This indicates that common method bias is not severe and remains within an acceptable range, suggesting that the study results are minimally affected by common method bias (44).

### 3.2 Physical activity level

At the physical activity level, there were 166 (36.7%; 78 males and 88 females) at the high intensity exercise level. For moderate intensity exercise level, there were 208 (46.0%; 54 males and 154 females). Low-intensity exercise level had 80 (17.7%; 30 males and 50 females). Male students at high intensity exercise level comprised 44.2% of the male group compared to 31.4% of the female group. Male students at medium intensity exercise level accounted for 31.4% of the male group, while female students accounted for 55.0%. Male students at the low intensity exercise level accounted for only 17.4% of the male group, while female students accounted for 17.9% (Table 1).

**Table 1:** Common method bias test.

Model	*χ^2^/df*	RMR	RMSEA	CFI	TLI
Original model	5.90	0.12	0.10	0.53	0.51
Two-factor model	4.56	0.11	0.09	0.63	0.60

### 3.3 Analysis of the effects of variations in trait anxiety, self-control, and physical activity on demographic factors

Using the independent samples t-test, differences in total scores for physical activity, self-control, and trait anxiety were examined by gender. The analysis’s findings revealed a substantial gender difference in physical activity (*p* < 0.001). Male university students (2839.42 ± 2315.47 METs-min/week) had higher physical activity total scores than female university students (2220.90 ± 2147.23 METs-min/week). The differences in self-control and trait anxiety were not significant by gender (see Table 2).

**Table 2:** Difference test for physical activity, self-control, and trait anxiety by gender (*M* ± SD).

Gender	PA (METs-min/week)	SC	TA
Male	2839.42 ± 2315.47	54.90 ± 10.96	54.53 ± 9.17
Female	2220.90 ± 2147.23	54.79 ± 10.69	53.12 ± 7.88
*t*	2.89^**^	0.11	1.74

^*^p<0.05; ^**^p<0.01; ^***^p<0.001.

The variations in total scores for physical activity, self-control, and trait anxiety among majors were examined using a one-way ANOVA test. Physical activity, self-control, and trait anxiety did not significantly differ among majors, according to the results, which are displayed in Table 3.

**Table 3:** Difference test for physical activity, self-control, and trait anxiety on major (*M* ± SD).

Major	PA(METs-min/week)	SC	TA
Science (*n* = 66)	2678.30 ± 2623.53	55.75 ± 10.92	44.97 ± 9.48
Engineering (*n* = 100)	2657.12 ± 2591.36	54.50 ± 10.34	45.92 ± 9.27
Agriculture (*n* = 40)	2721.30 ± 1929.18	54.50 ± 10.82	50.10 ± 7.46
Medicine (*n* = 80)	2402.70 ± 2103.62	54.90 ± 12.87	46.95 ± 8.96
Social Sciences (*n* = 166)	2208.93 ± 1972.76	54.69 ± 10.13	45.69 ± 8.29
*F*	0.51	0.08	1.31

PA, Physical activity; TA, Trait-anxiety; SC, Self-control.

The variations in total scores for physical activity, self-control, and trait anxiety between grades were examined using a one-way ANOVA test. According to Table 4, there were no discernible variations in physical activity, self-control, or trait anxiety across grades.

**Table 4:** Difference test of physical activity, self-control, and trait anxiety on the grade level (*M* ± SD).

Grade	PA(METs-min/week)	SC	TA
Freshman (*n* = 130)	2418.74 ± 2347.34	54.55 ± 11.72	46.54 ± 9.60
Sophomore (*n* = 136)	2459.49 ± 1843.80	54.94 ± 9.96	46.60 ± 7.96
Junior (*n* = 72)	2284.33 ± 2242.40	57.16 ± 11.56	45.75 ± 7.75
Senior (*n* = 24)	4018.00 ± 2496.57	49.75 ± 7.08	43.42 ± 11.90
Post-graduate (*n* = 90)	2226.69 ± 2450.38	54.53 ± 10.67	46.47 ± 8.75
*F*	1.66	1.11	0.39

### 3.4 Analysis of the relationships between trait anxiety, self-control, and physical activity

A Pearson correlation analysis was conducted on physical activity, self-control, and trait anxiety. The results are shown in Table 5, which indicates that physical activity, self-control, and trait anxiety are significantly correlated (*p* < 0.01).

**Table 5:** Analysis of means, standard deviations, and correlation coefficients for variable (*n* = 452).

Variable	*M*	SD	PA	SC	TA
PA	2456.27	2230.57	1		
SC	54.83	10.78	−0.56^**^	1	
TA	53.66	8.41	−0.56^**^	0.53^**^	1

PA, Physical activity; TA, Trait-anxiety; SC, Self-control; ^*^p < 0.05, ^**^p < 0.01, ^***^p < 0.001.

### 3.5 Mediation test for trait anxiety

**Figure 1:**
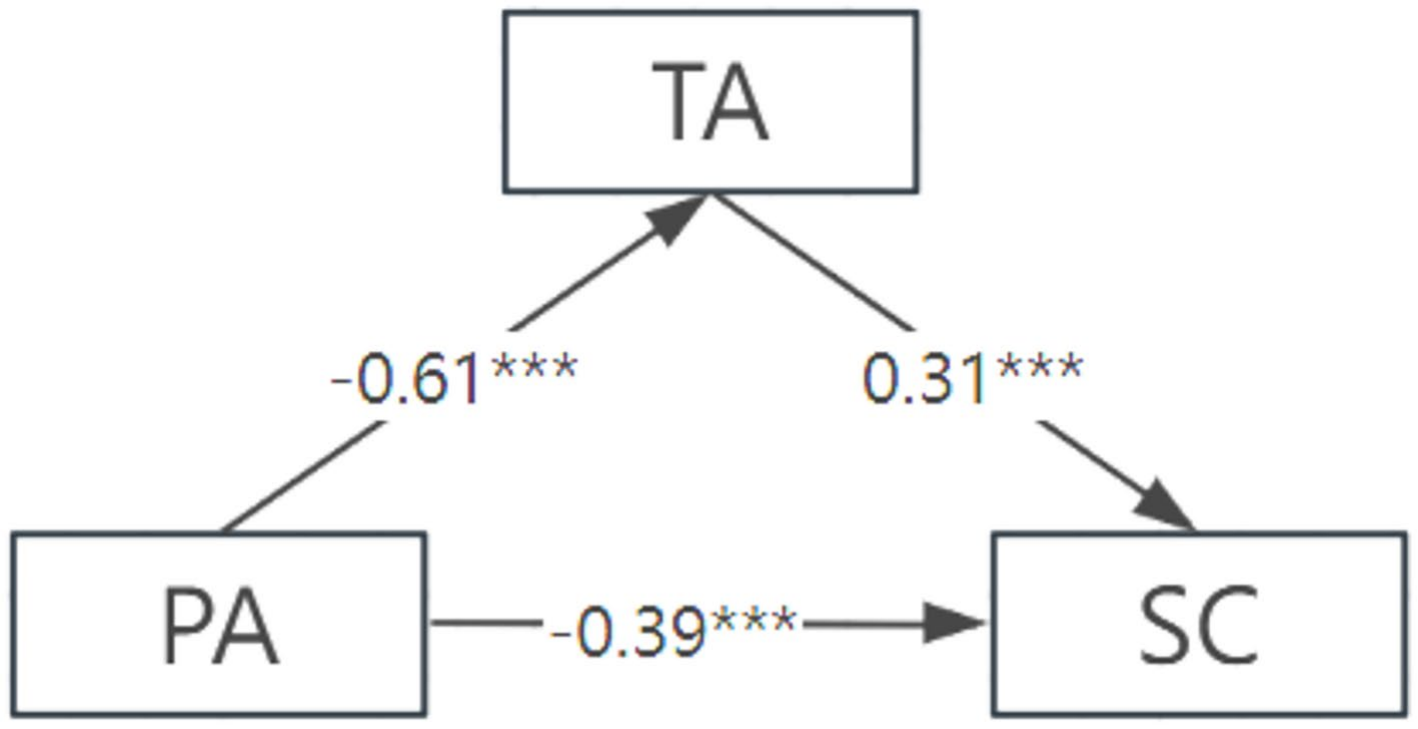
Mediation effect testing path. ***p < 0.001.

We used Model 4 in the Process 4.1 plugin to test the mediating role of trait anxiety. Physical activity significantly and negatively predicted trait anxiety (*β* = −0.61, *t* = −14.61, *p* < 0.001). After including trait anxiety in the regression equation, trait anxiety significantly and positively predicted low self-control (*β* = 0.31, *t* = 6.88, *p* < 0.001), and physical activity still significantly and negatively predicted low self-control (*β* = −0.39, *t* = −8.05, *p* < 0.001), as shown in Table 6. This indicates that trait anxiety partially mediates the relationship between physical activity and self-control, as shown in Figure 1.

**Table 6:** Tests of mediating effects of trait anxiety between physical activity and self-control.

Result variables	Predictive variables	*R*	*R^2^*	*F*	*β*	*t*	95% CI
TA	PA	0.58	0.33	55.33***	−0.61	−14.61***	[−0.69, −0.52]
	Gender				−0.04	−1.37	[−0.09 0.02]
	Major				−0.02	−0.07	[−0.07, 0.03]
	Grade				0.15	2.62**	[0.04, 0.26]
SC	PA	0.62	0.38	54.86***	−0.39	−8.05***	[−0.49, −0.30]
	TA				0.31	6.88***	[0.22, 0.41]
	Gender				0.01	0.20	[−0.05, 0.05]
	Major				0.00	0.06	[−0.05, 0.05]
	Grade				0.04	0.74	[−0.07,0.15]

PA, Physical activity; TA, Trait-anxiety; SC, Self-control; EIL, Exercise intensity level; ^*^p < 0.05, ^**^p < 0.01, ^***^p < 0.001.

The analysis results were confirmed one after the other, and the mediating impact was further tested using the bootstrap approach (5,000 extractions, 95% confidence level). Table 7 displays the results, and there are no zeros in the confidence intervals for each path. This shows that all of the standardized effect values are significant and that all of the direct, indirect, and total impacts are statistically significant. According to the aforementioned findings, trait anxiety has a statistically significant mediation role between self-control and physical activity. The mediating effect of trait anxiety accounted for 32.76 [(−0.19)/ (−0.58) *100%] of the overall effect of physical exercise on self-control, which was −0.58 [(−0.39) + (−0.19)].

**Table 7:** Dissection of the overall, direct, and mediating effects.

Parameter		Effect	SE	95% CI		Effect percent
				LLCI	ULCL	
Total effect	PA → SC	−0.57***	0.04	−0.65	−0.49	
Direct effect	PA → SC	−0.39***	0.05	−0.48	−0.30	68.42%
Indirect effect	PA → TA → SC	−0.18***	0.03	−0.24	−0.12	31.58%

### 3.6 Test of the moderating effect of gender

**Figure 2:**
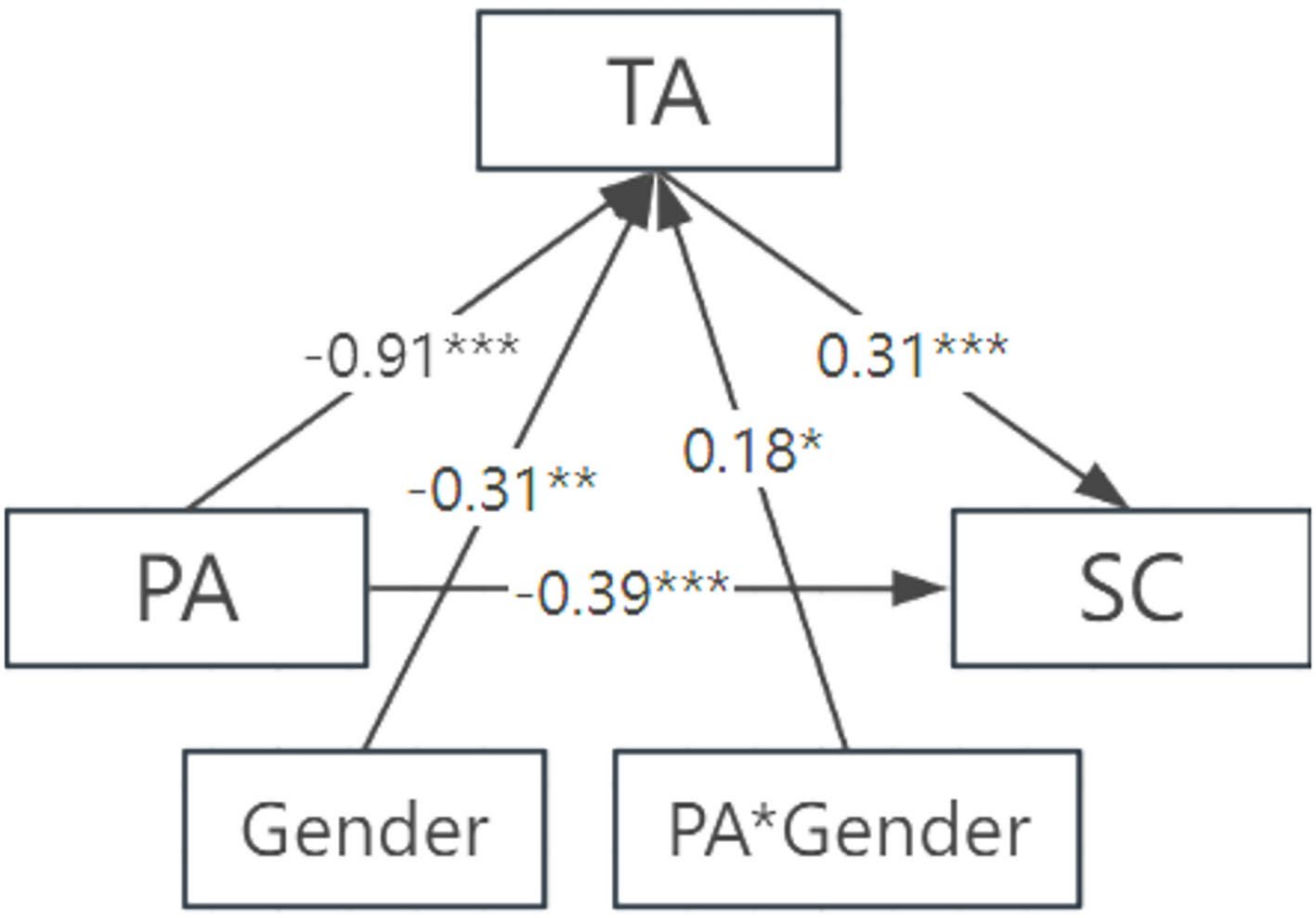
Testing pathway for moderating effects. *p < 0.05, **p < 0.01, ***p < 0.001.

We used Model 7 in the Process 4.1 plugin to test the moderating effect of gender. The results show that physical activity (*β* = −0.91, *p* < 0.001) and gender (*β* = −0.31, *p* < 0.001) significantly and negatively predict trait anxiety, while the interaction term between physical activity and gender significantly and positively predicts trait anxiety (*β* = 0.18, *p* = 0.02), as shown in Table 8 and Figure 2.

**Table 8:** Analysis of moderating effects.

Result variables	Predictive variables	*R*	*R^2^*	*F*	*β*	*t*	95% CI
TA	PA	0.60	0.36	41.44^***^	−0.91	−6.94^***^	[−1.16, −0.65]
	Gender				−0.31	−3.81^**^	[−0.47, −0.15]
	PA*Gender				0.18	2.36^*^	[0.03, 0.33]
	Speciality				−0.01	−0.20	[−0.06, 0.04]
	Grade				−0.03	−1.06	[−0.08, 0.02]
	EIL				0.13	2.23^*^	[0.02, 0.24]
SC	PA	0.62	0.38	68.51^***^	−0.39	−8.05^***^	[−0.49, −0.30]
	TA				0.31	6.87^***^	[0.22, 0.40]
	Speciality				0.01	0.20	[−0.05, 0.05]
	Grade				0.00	0.06	[−0.05, 0.05]
	EIL				0.04	0.74	[−0.07, 0.15]

PA, Physical activity; TA, Trait-anxiety; SC, Self-control; ^*^p < 0.05, ^**^p < 0.01, ^***^p < 0.001.

**Figure 3:**
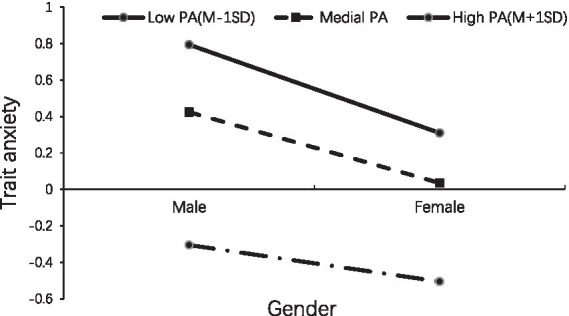
Plot of the simple slope test for moderating effects by gender. PA: physical activity.

A simple slope analysis was conducted for different gender groups. The results showed that the predictive effect of physical activity on trait anxiety was stronger among male college students (simple slope = −0.72, *p* < 0.001) than among female college students (simple slope = −0.54, *p* < 0.001). Subsequently, the mediating role of trait anxiety was tested for college students of different genders. The results showed that among male college students, the mediation effect of trait anxiety was −0.23 [95% CI: (−0.31, −0.14)], and the mediation effect was significant. Among female college students, the mediation effect of trait anxiety was −0.17 [95% CI: (−0.21, −0.11)], and the mediation effect was significant, as shown in Table 9 and Figure 3.

**Table 9:** Analysis of the moderating effect of gender.

Model	Career calling	Effect	SE	95%[CI]
PA-gender	Male	−0.69^***^	0.06	[−0.84, −0.60]
	Female	−0.51^***^	0.05	[−0.64, −0.44]
Mediation effect	Male	−0.23	0.02	[−0.31, −0.14]
	Female	−0.17	0.01	[−0.22, −0.11]

PA, Physical activity; *p < 0.05, **p < 0.01, ***p < 0.001.

## 4 Discussion

This study aims to investigate the underlying mechanisms through which physical activity influences self-control among college students, with a particular focus on the mediating role of trait anxiety and the moderating role of gender. Consistent with our hypotheses, we found that higher levels of physical activity significantly enhance self-control among college students, and this effect is partially mediated by reduced levels of trait anxiety. Furthermore, the first half of this mediating pathway (physical activity → trait anxiety) is moderated by gender, indicating that the “stress-reducing” effect of physical activity is more pronounced among male students. The following sections will provide a detailed discussion of the main findings.

### 4.1 The relationship between physical activity and self-control

The results indicate a significant negative correlation between physical activity and low self-control, meaning that physical activity significantly and positively predicts self-control among college students. This finding, which supports Hypothesis 1, is consistent with previous research (19, 20). This finding can be further explained from the perspective of the strength model of self-control. This model likens self-control resources to “muscle strength,” which is both finite and trainable (23). For college students who maintain a long-term exercise routine, each workout represents a process of self-control in which they actively overcome internal impulses and external temptations. Although this process depletes immediate self-control resources, consistent and regular engagement acts as repeated “resistance training” for the “self-control muscle.” Such training effectively raises the “baseline level” or “maximum capacity” of self-control resources, providing individuals with a more abundant reserve of resources when facing future challenges and thereby fundamentally enhancing their self-control abilities. Therefore, physical activity is not merely a drain on self-control resources but rather a proactive, long-term investment that can fundamentally shape and enhance college students’self-control abilities.

### 4.2 The mediating role of trait anxiety

The results indicate that trait anxiety partially mediates the relationship between physical activity and self-control, thereby confirming Hypothesis 2. This finding enriches previous path analysis studies on the effects of physical activity on self-control and expands the Anxiety-Group Activity Model (45), suggesting that while trait anxiety influences an individual’s physical activity, physical activity also influences trait anxiety levels.

First is the direct pathway through which physical activity influences self-control. Even after controlling for the effects of trait anxiety, physical activity still significantly predicts self-control. This is consistent with the self-control resource model. Long-term physical activity directly enhances an individual’s total self-control resources or the efficiency with which they are utilized—much like “muscle training”—thereby improving self-control ability. This is a direct, bio-psychological “capacity-building” process.

Second is the indirect pathway through which physical activity influences self-control via trait anxiety. Increased physical activity significantly reduced trait anxiety levels among college students, and lower trait anxiety, in turn, predicted stronger self-control. This indirect pathway can be explained by combining the multidimensional anxiety theory and the self-control resources model. According to the multidimensional anxiety theory, anxiety comprises two components: physical tension and cognitive worry (31). Physical activity, on the one hand, consumes excess stress energy and reduces physiological tension; on the other hand, it diverts attention and interrupts catastrophic thinking about negative events, thereby systematically lowering an individual’s baseline anxiety level. According to the self-control resource model, an individual’s cognitive resources are limited (23). In individuals with high trait anxiety, a portion of their cognitive resources is occupied by persistent, diffuse worries and negative emotions. When this portion of resources is reduced, individuals can free up more cognitive resources to perform active self-control tasks. Thus, physical activity indirectly “frees up” and “conserves” critical cognitive resources for self-control by reducing trait anxiety. This allows individuals to allocate more cognitive resources to self-control tasks, thereby enhancing their self-control performance. In summary, physical activity maintains an indirect relationship between trait anxiety and self-control. This suggests that reducing trait anxiety levels in college students can promote the development of their self-control abilities and, consequently, support their mental health.

### 4.3 The moderating role of gender

The results indicate that gender moderates the relationship between physical activity and trait anxiety, thereby confirming Hypothesis 3. This finding enriches the existing research on the relationship between physical activity and trait anxiety. The findings suggest that, compared to female college students, physical activity has a stronger predictive effect on trait anxiety among male college students. Furthermore, trait anxiety exerts a stronger mediating effect on the relationship between physical activity and self-control. This may be attributed to the differing sociocultural influences on gender. Traditional sociocultural norms encourage men to “act rather than speak,” leading them to prefer physical activity as a means of venting emotions and diverting attention when faced with stress, rather than confiding in others or seeking emotional support (44). Consequently, physical activity serves as a more central and effective anxiety-regulation strategy for men. Women, on the other hand, may rely more on strategies such as social sharing, emotional expression, or cognitive rumination. Physical activity holds relatively less weight in their coping toolkit (46). Furthermore, failures in self-control among men are more closely associated with impulsive behavior, whereas such failures among women are more closely linked to difficulties in emotional regulation (48). Therefore, for men, reducing trait anxiety may be a key mediating pathway for improving self-control. For women, however, even with reduced anxiety levels, improvements in self-control may still depend on other mediators (such as improved emotional regulation strategies or social support), thereby diluting the specific mediating effect of trait anxiety.

## 5 Limitations and perspectives

Inevitably, this study also has some limitations. First, as this study employed a cross-sectional design, it was unable to clarify the causal relationships among the variables. Future research could incorporate a longitudinal design to further examine the directionality and developmental trends of these variables. Second, this study found that trait anxiety partially mediates the relationship between physical activity and self-control. This suggests that other factors may also influence the relationship between physical activity and self-control, and future research could explore these potential factors. Finally, this study primarily used the total scores of each scale for mediation analysis to examine the overall effect of physical activity on self-control. However, both self-control and trait anxiety are multidimensional constructs, and their different dimensions may exhibit distinct effects within the pathway of physical activity’s influence. Future research could further explore the specific mechanisms by which the various dimensions of self-control (such as impulse control and healthy habits) and the various dimensions of trait anxiety operate within the relationship between physical activity and self-control, thereby providing a more detailed understanding of the mediation pathways identified in this study.

## 6 Conclusion

1. Physical activity is a significant predictor of self-control among college students. College students with higher levels of physical activity also exhibit higher levels of self-control.
2. Trait anxiety partially mediates the relationship between physical activity and self-control.
3. Gender moderates the relationship between physical activity and trait anxiety.

## Data Availability

The raw data supporting the conclusions of this article will be made available by the authors, without undue reservation.
